# Reply: Aspirin for Secondary Prevention: Are Patients Getting Wiser Than Their Physicians?

**DOI:** 10.1016/j.jacadv.2025.102309

**Published:** 2025-10-29

**Authors:** Karthik Murugiah, Claudia See, Chenxi Huang

**Affiliations:** a Section of Cardiovascular Medicine, Department of Internal Medicine, Yale School of Medicine, New Haven, Connecticut, USA; b Center for Outcomes Research and Evaluation, Yale-New Haven Hospital, New Haven, Connecticut, USA; c Department of Medicine, University of California, San Francisco, California, USA

We thank Dr Cleland and colleagues for their insightful reply to our recent *JACC: Advances* paper^1^ highlighting the inconsistencies between evidence, guidelines, and practice in the use of aspirin for cardiovascular prevention.

With recent negative trials, the use of aspirin for primary prevention has decreased. The decrease from 21% to 16%, however, is not as precipitous as one would like. We know that there remains a lag from evidence to practice in medicine. It cannot be stressed enough that evidence is only as good as its implementation. Health systems and physicians need to be nimble and be able to react to emerging evidence by implementing new therapies and de-escalating harmful ones.

The decline in aspirin for secondary prevention was smaller—66% to 62%. We posited that these small declines may be from increasing single agent P2Y12 use after revascularization, and aspirin discontinuations among anticoagulated patients, both supported by guidelines. However, outside of these situations guidelines continue to endorse aspirin long-term for secondary prevention. The authors correctly point out that the evidence for aspirin, let alone long-term, is less robust than is sometimes assumed. The evidence for the individual benefit of aspirin rests on cumulative evidence from myocardial infarction trials conducted in earlier eras, before the widespread adoption of therapies that have since become standard of care—such as statins and routine revascularization. In this modern landscape, the benefit of aspirin is less well defined.

Although we and the authors concur on this issue, opinions can only stir debate, and evidence alone can bring transformation. Evidence is now robust for aspirin discontinuation after revascularization following a month of dual antiplatelet therapy. A recent trial explored aspirin discontinuation even sooner with potent P2Y12s but results were mixed with increased ischemic events but lower bleeding.^2^ Single P2Y12 after a short period of dual antiplatelet therapy will likely become standard practice after revascularization. However, what should the strategy be after the routine P2Y12 therapy duration—long-term P2Y12—which can have cost implications, long-term aspirin, or no antiplatelets? There is an urgent need for trials in this regard. Reassessing aspirin’s role in conditions like stroke and peripheral arterial disease is also needed.

The authors suggested that medical skepticism could be contributing to the reduction in secondary prevention aspirin use. Certainly possible, but to digress from this specific point, the larger issue is the rising medical skepticism among patients.^3^ With the increasing politicizing of medicine and rising misinformation in social media, trust in the medical system is under jeopardy. To maintain trust in medicine researchers must continually ask the right questions and generate high-quality evidence, and practicing physicians and health systems must remain primed to react to such evidence. It was heartening to see with the series of recent trials on beta blockers after myocardial infarction that the cardiovascular fraternity is prepared to question long-held beliefs.
